# Comparison between Virtual and Traditional Learning Methods for Orthodontic Knowledge and Skills in Dental Students: A Quasi-Experimental Study

**DOI:** 10.3390/healthcare9091092

**Published:** 2021-08-24

**Authors:** Zaki Hakami

**Affiliations:** Division of Orthodontics, Department of Preventive Dental Sciences, College of Dentistry, Jazan University, Jazan 45142, Saudi Arabia; zhakami@jazanu.edu.sa

**Keywords:** online learning, virtual learning, traditional learning, undergraduate education, orthodontics, dental education, orthodontic knowledge, orthodontic skills, Blackboard

## Abstract

A gradual transition from traditional face-to-face learning to online learning has been observed globally following the COVID-19 pandemic. The aim of the study was to compare online and traditional learning methods in relation to orthodontic knowledge and skills acquired in undergraduate courses. A quasi-experimental design was used with two consecutive year classes of 198 dental students at Jazan University, Saudi Arabia. The experimental (virtual) group of 92 students received the content of a clinical orthodontic course virtually using the Blackboard Collaborate™ platform. The control (traditional) group consisted of a previous cohort of 106 students who enrolled in the same course but received traditional classroom education. The students were evaluated through a semester, and both groups obtained the same content and exam questions. The students in the virtual group scored higher in the final theory exam than the traditional group (*p*-value < 0.05). However, no significant difference in the overall orthodontic knowledge and skills was observed between the groups at the end of the course. Both learning methods showed moderate positive correlation between orthodontic knowledge and skills. In conclusion, virtual learning can serve as an effective alternative to traditional face-to-face learning for teaching orthodontic knowledge and skills to the dental students.

## 1. Introduction

It has been one hundred years since dentistry became a specialty. As dentistry evolved in the era of physics and chemistry, the initial research focused on improving materials and techniques [1]. Since the 18th century, orthodontics has been a recognized specialty. Unlike other branches that seek to ameliorate diseases, orthodontics is a different academic discipline [2]. The process of obtaining orthodontic competency is very important. Therefore, an objective competency-based course in this area has the potential to validate the product of education, create consistency in predoctoral orthodontic education, and provide practitioners with the necessary skills to manage patients. Moreover, dental educators are searching for efficient and effective ways to promote learning and retention of knowledge and skills [3,4].

Since the World Health Organization declared the coronavirus disease 2019 (COVID-19) outbreak a pandemic on 11 March 2020, many countries enforced lockdowns and quarantine measures to curb its spread. Consequently, there has been a general shift from traditional face-to-face instruction to online learning. Online learning/electronic (e)-learning is a type of learning environment that takes place over the internet where knowledge can be transferred and acquired virtually using multiple medias. Accessibility to information despite location, a more customized learning experience, cost-effectiveness, improved scope of learning as per individual interest, comfort and flexibility of sharing knowledge with others are some of the important benefits of this [5]. As a result, professors and students at various universities began to explore and access the academic cyber world, which helped them to communicate with guidance in simulated environments. The willingness and acceptance of students to make greater use of online learning to obtain a standard education remain unclear [6].

In contrast to face-to-face education, the emergence of online education has led to student-centered approaches, in which the students can self-regulate what they study [7]. Massive open online courses in medical education have shown comparable results to existing on-campus courses [7,8]. A recent systematic review reported that virtual learning was found to be effective among medical students during the COVID-19 crisis [9]. Students have been able to keep up with the newest medical advances due to open-access guidance from medical experts, and they were also able to retrieve the knowledge lost due to suspension of classes and clinical postings [9]. Few studies suggested that e-learning can be used successfully in a dental school’s curriculum to enhance students’ learning, especially in the clinical curriculum [10,11,12,13]. 

Several studies have suggested that online and blended educational approaches can be compared to those of traditional classroom models [14,15]. The observed advantages of online learning over traditional methods in teaching the undergraduate orthodontic curriculum are contradictory [16,17,18]. Most of the studies focusing on this topic targeted theoretical education. Few studies have made comparisons between the effect of e-learning and traditional learning on teaching disciplines, where manual dexterity is required [19,20]. No significant difference in orthodontic practical skills was found between lectures and video demonstrations [20,21]. In addition, the efficacy of virtual education for teaching orthodontic diagnosis and treatment planning has received little attention.

In Saudi Arabia, a study on dental students’ perceptions of online learning conducted at King Saud University showed overall positive responses regarding the usefulness of online tutorials. Moreover, few students supported the replacement of traditional lectures and live demonstrations with online tutorials, while most students preferred a combination of these teaching methods [22]. There is a scarcity of studies focused on the effectiveness of online learning among students by assessing their marks and evaluating academic performance following this pandemic crisis. Therefore, this study was undertaken to assess the difference in orthodontic knowledge and skills among undergraduates gained from the traditional face-to-face learning and online learning methods. Moreover, gender variations in acquiring orthodontic knowledge and skills were evaluated.

## 2. Methods

The study was conducted among the sixth-year dental students of two consecutive years at Jazan University, College of Dentistry, to compare orthodontic knowledge and skills in undergraduate courses between virtual and face-to-face traditional learning methods. The study was reviewed and approved by the Ethical Committee of Scientific Research Unit, College of Dentistry, Jazan University (Reference Number: CODJU-2014F).

### 2.1. Study Procedure

A quasi-experimental design was used in which a retrospective control group in the first semester of the 2019 academic year was compared to a prospective experimental group in the first semester 2020. This study used convenient sampling, and all selected students were enrolled in a two-credit clinical orthodontic I course with three contact hours per week (1 h for lectures and 2 h for practical sessions). The clinical orthodontic I course is a 15 week-long course taught in the first semester of the last year of the six-year program of Bachelor of Dental Surgery (BDS) and includes the course learning outcomes indicated in Table 1. The students in the control group were taught the course using face-to-face traditional lecturing method consisting of didactic PowerPoint presentations in the classrooms along with a practical demonstration of the orthodontic appliances and instruments at the phantom lab (traditional group). By contrast, the students in the experimental group obtained the theoretical and the practical education through fully virtual learning via the Blackboard Collaborate™ platform (Blackboard Inc., Reston, VA, USA) following the COVID-19 pandemic (virtual group). Both groups were taught by the same instructors using the same references and contents.

The Blackboard Collaborate™ is an e-learning platform and a learning management system that provides various interactive learning tools, such as announcements, calendars, tasks, assessments and grading [23]. The mentor uploads the re-prepared content of the lectures and then streams live video with the students. In the practical sessions, the students were divided into four groups. Recorded videos for the demonstration of orthodontic appliances and instruments were uploaded onto Blackboard. The students could interact with the presenter and asked questions through the discussion panel or by talking via their microphones. The students were given weekly assignments. All virtual sessions were recorded, and the presentations files were uploaded into the course content on Blackboard. Moreover, the students were provided with online books, and all course materials were available anywhere and anytime. 

### 2.2. Outcome Assessment

The types of educational assessments in the course were midterm and final theory examinations, objective structured clinical examination (OSCE), and a spotter examination. The midterm and final theory examinations included multiple-choice questions, whereas OSCE and the spotter examination included short essay questions. The midterm examination covered learning outcomes 1.1 and 1.2 and the final theory examination covered learning outcomes 1.2, 1.3, 2.1 and 2.2 (Table 1). OSCE covered learning outcomes 2.1 and 2.2 (Table 1), and the students were asked to diagnose and formulate a treatment plan from orthodontic records photos. In the spotter examination, the students were asked to identify the orthodontic appliances and instruments and determine their uses, which covered learning outcome 2.3 (Table 1).

The level of knowledge among the students was assessed by combining the scores obtained in the midterm and final theory examinations. Skills acquired among the students were assessed by combining the scores of OSCE and spotter examination. In both groups, the students received the same type of evaluation methods and questions. The questions of the exams were reviewed by the exam review committee at the Preventive Dental Sciences Department, College of Dentistry, Jazan University. 

### 2.3. Data Management and Analysis

Data were analyzed using SPSS version 26 (IBM Corporation, Armonk, NY, USA). A chi-squared test was used to compare the descriptive characteristics of the variables, grade point average (GPA) and the gender distribution across traditional and online learning. All variables were tested for normality using the Shapiro–Wilk test to identify any differences among the two groups. Overall differences in the course learning outcomes, including knowledge and skills, between the traditional and virtual learning methods were compared using the Mann–Whitney test. Moreover, the Spearman rho test was used to assess the correlation between the scores and the assessment methods. The *p*-value was set at 0.05 for all tests.

## 3. Results

A total of 198 students with a median age of 23 years (IQR: 23–24) were enrolled in the course of two consecutive years, of whom 106 students attended the traditional learning and 92 students attended the virtual learning. There was no statistical difference in terms of the students’ number and age between the groups. Males (53.54%) constituted the majority of the study students with no significant difference between the number of males and females and their distribution in traditional and virtual learning groups. The median GPA score in the virtual group was significantly higher than that in the traditional group (4.35 (IQR: 4.02–4.55) and 4.14 (IQR: 3.67–4.47), respectively) as shown in Table 2.

In general, there was a significant, moderate correlation (0.667, *p* < 0.0001) between the orthodontic knowledge and skills when the students were taught via either virtual or traditional learning methods. No statistically significant difference was found in the level of knowledge and skills between the groups. However, slightly higher knowledge and skill scores were observed in the students in the virtual learning group compared to those in the traditional group (Table 3). 

Table 4 shows a comparison of the median scores acquired by students in the two groups with respect to gender and the assessment methods. The median score acquired by students in the traditional group in the midterm exam was higher than that in the virtual group (*p*-value < 0.05). However, the students in the virtual group scored higher in the final theory exam than the traditional group. No significant difference was noted between the two groups regarding the scores acquired on the OSCE and spotter exam (Table 4). 

In the virtual group, the students with high GPAs scored better in the spotter exam. Besides this, both learning methods showed a significant strong correlation between knowledge and GPA and a moderate correlation between skills and GPA (Table 5). 

Concerning gender differences, mean scores classified by gender, learning and assessment method are shown in Table 6. A statistically significant difference was found within the online group between male and female students, as females showed better outcome scores in the final theory exam than those of males.

## 4. Discussion

Most educational institutions temporarily closed following the spread of the COVID-19 pandemic. As a result, the only way to continue to provide education was via digital technology. Online learning is receiving progressive popularity in the field of dental education. This quasi-experimental study demonstrated that the virtual learning method is comparable to the traditional one in teaching orthodontic knowledge and skills in undergraduate dental courses. The results of the current study were consistent with those of other studies in the medical field, where online learning can be considered a potential method in undergraduate medical teaching [24]. Moreover, the provision of courses in other dental disciplines in a web-based format as a supplement to traditional lectures was found to be an overall success [25].

The COVID-19 pandemic has shown that digital technologies will play a dominant role in the future development of dental education [26]. Some studies emphasized that online learning can be considered as an effective method of learning, and various institutions are working to further develop these resources to improve student engagement and interactivity [26]. A systematic review reported that e-resources were a useful supplement to traditional lectures, and e-modules enhanced the learning experience of orthodontic students [27]. The results of this study complement a study conducted by Ludwig et al., who found that e-learning was effective in teaching cephalometry in orthodontics. All of the students using e-learning methods were able to identify the cephalometric anatomical landmarks precisely [28]. Furthermore, Mehta et al. reported that e-resources were a useful supplement to traditional lectures, and e-modules enhanced the learning experience of orthodontic students. Distance learning in orthodontics was proven to be effective with no significant difference from the traditional learning method [29]. 

Several survey studies during the pandemic have reported either positive or negative attitudes toward online teaching among different dental colleges in different countries [30,31]. The dental students in Saudi Arabia have considered online learning helpful as a supplement to their learning rather than a replacement for traditional teaching methods [22]. In this study, however, the method of education did not affect the overall knowledge obtained at the end of an orthodontic course. Moreover, a temporal adaption to the online environment was observed, as the students in the online learning group achieved lower scores in the midterm exam but higher scores in the final exam compared to the students in the traditional group. Similar findings were reported in other dental disciplines where the mean knowledge scores of Iranian dental students who received virtual learning were higher than those of students who had obtained traditional learning in endodontic and radiology courses [32]. 

In addition to theoretical education, dental students must develop the clinical skills necessary to diagnose a patient’s problems and provide a suitable treatment plan [33]. Abbasi et al. conducted a study among health science students regarding the perception and satisfaction of e-learning in which most of the students agreed that an e-learning platform was not useful for developing clinical and technical skills [34]. Similarly, another study reported that most of the dental and medical students agreed that online classes were not as effective as classroom classes [35]. However, further studies are also needed in various other specialties to fully understand the impact of e-learning on students’ performance, including examinations and clinical competency outcomes.

Gormley et al. reported that undergraduate medical students considered e-learning to be just as effective as other traditional methods for the teaching of clinical skills [36]. In the current study, the dental students developed sufficient clinical skills for diagnosis and treatment planning orthodontic cases irrespective of the learning methods. These results support the findings that e-learning and video demonstrations have only minor effects on orthodontic practical skills [20]. Furthermore, the results of this study are in alignment with the literature reporting that student achievement is as high in the virtual environment as it is when delivered in a traditional face-to-face classroom for providing education regarding radiographic interpretation of bony lesions of the jaw [37].

Gender differences in academic performance and self-efficacy in online-learning environments were documented. The results of our study are in line with the general trend that female students perform better than males on the online courses [38]. Although easy access to online materials creates more satisfaction among students [39], their levels of interest in the subject decreases the degree of motivation and satisfaction among them, in addition to the unavailability of well-equipped infrastructure [14,37]. In the current study, the students’ satisfaction was not measured. The relationship between the level of satisfaction and the observed effects on the students’ performance and gender differences would provide more insights into the teaching methods.

The Blackboard system provides a useful educational environment where students can interact socially and academically with each other and staff [40]. It also has some limitations, such as it does not ensure the real attendance and the focus of students during the lectures [41]. In general, studies have shown a contradictory relationship between class attendance and academic performance of students [42,43]. This could be evident with access availability to the educational contents and the recorded lectures at any time in online learning. While online learning is an opportunity to learn without restrictions due to geographical constraints and provides a good option for lifelong learning, it also comes with its share of challenges and obstacles that obstruct its effective usage [44].

In this study, students were evaluated for the entire semester using routine assessment methods. This is a strength of the study, as the risk of bias related to the Hawthorne effect is neglected. However, some limitations related to the nature of a quasi-experimental study design should be acknowledged. Randomization and pure experimental design were not possible in obligatory university courses; nevertheless, two-consecutive-year students with similar demographics in the groups would reduce the selection bias.

Moreover, another limitation of this study was that the learning methods were evaluated on final-year students. As the knowledge varies between academic years, using senior students could lead to bias in the study because of their knowledge and skills in orthodontics gained from other disciplines [20]. Therefore, future multi-center studies with large sample sizes to compare the effectiveness of traditional versus online learning of both theoretical and practical subjects would lead to definitive conclusions.

## 5. Conclusions

Based on the findings of the study, I can conclude that there was no significant difference in orthodontic knowledge and skills among students irrespective of the learning methods. However, higher knowledge and skill scores were obtained for those who had received online learning. The female students in the virtual group scored higher in the final theory exam than the male students. In view of the advantage of online methods for teaching theoretical topics and its equal effectiveness in training clinical skills, it can serve as an effective alternative to traditional face to face learning.

## Figures and Tables

**Table 1:** Clinical orthodontic I course learning outcomes.

Course Learning Outcomes	
1	Knowledge and Understanding: By the end of the course, the student will be able to:
1.1	Describe the biology and mechanics of tooth movement.
1.2	Discuss the various types of anchorage in orthodontics.
1.3	Describe the fundamentals of identification and management of different malocclusions and dental and skeletal discrepancies.
2	Skills: By the end of the course, the student will be able to:
2.1	Interpret the findings and make a proper diagnosis of various malocclusions.
2.2	Formulate an appropriate treatment plan for a specific malocclusion.
2.3	Differentiate between the various components of orthodontic appliances and orthodontic instruments and summarize their uses.
2.4	Manipulate an orthodontic wire to construct basic components of simple removable appliances.
3	Values: By the end of the course, the student will be able to:
3.1	Demonstrate punctuality, ethical behaviors and professional attitudes

**Table 2:** Descriptive statistics of types of educational assessments in orthodontic course.

**Category**		**Traditional Group (N)**	**Virtual Group (N)**	**Total (N)**
Gender	Males	47	50	97
	Females	59	42	101
	Both	106	93	198
**Category**		**Traditional Group** **Median (IQR)**	**Virtual Group** **Median (IQR)**	**Total** **Median (IQR)**
Age		23 (23–23)	23 (23–24)	23 (23–24)
GPA *		4.14 (3.67–4.47)	4.35 (4.02–4.55)	4.23 (3.81–4.52)
Midterm		11.25 (10.5–12.25)	10.50 (9.00–12.00)	11 (9.50–12.25)
OSCE		6.5 (5.5–7.5)	6.75 (5.50–8.00)	6.50 (5.50–7.50)
Final spotter		7.50 (6.5–9)	8.30 (7.22–8.89)	8 (6.75–8.89)
Final theory		22.5 (20–25)	24 (22.00–26.50)	23 (20.50–25.50)
General average score		48.38 (41.75–52.50)	48.58 (43.89–53.22)	48.53 (42.50–53.06)

N: number; IQR: interquartile range; * chi-squared test is significant at *p* < 0.05.

**Table 3:** Comparisons between level of knowledge and skills among students.

	Traditional Group	Virtual Group	*p*-Value
	Median (Range)	Median (Range)	
Knowledge	33.25 (23)	34.5 (23.5)	0.230
Skills	14.25 (11.75)	14.61 (11.61)	0.400

Mann–Whitney test is significant at *p* < 0.05.

**Table 4:** Difference in scores of students in terms of gender and evaluation type.

	Gender			Evaluation Type		
	Median (Range)			Median (Range)		
	Overall	Males	Females	Overall	Traditional Group	Virtual Group
Midterm	11 (10)	10.75 (10)	11.03 (11.25)	11 (10) *	11.25 (9) *	10.5 (10)
OSCE	6.5 (8)	6.5 (8)	6.5 (8)	16 (27)	6.5 (8)	6.75 (8)
Spotter	8 (7.5)	8 (5.5)	7.8 (7.5)	8 (7.5)	7.5 (7.5)	8.33 (4.44)
Final theory	23 (17.5) *	23 (13.5)	23.5 (17.5) *	18.5 (23) *	22.5 (17)	24 (14) *

* Mann–Whitney test is significant at *p* < 0.05.

**Table 5:** Correlation of GPA and performance level between the groups.

	GPA	
	Traditional Group (R)	Virtual Group (R)
Knowledge	0.81	0.83
Skills	0.62	0.69
Midterm	0.71	0.67
OSCE	0.52	0.50
Spotter	0.52	0.70
Final Theory	0.75	0.81

(R): correlation coefficient. Spearman rho test is significant at *p* < 0.001 at all readings.

**Table 6:** Comparison between male and female performance in course tests among the groups.

	Traditional Group		Virtual Group	
	Males (*n* = 47)	Females (*n* = 59)	Males (*n* = 50)	Females (*n* = 42)
Midterm	11 ± 1.82 ^a^	11.25 ± 1.77	10.18 ± 2.35	10.72 ± 2.23
OSCE	6.55 ± 1.50	6.40 ± 1.51	6.33 ± 1.75	6.80 ± 1.60
Spotter	7.8 ± 1.43	7.64 ± 1.88	7.9 ± 1.06	7.91 ± 1.01
Final theory	22.02 ± 3.15	22.35 ± 3.67	22.93 ± 3.22 *	24.29 ± 3.27

^a^: mean ± standard deviation. * Mann–Whitney test is significant at *p* < 0.05.

## Data Availability

The data will be provided upon request.
